# Quality evaluation of rapeseed oil in Chinese traditional stir‐frying

**DOI:** 10.1002/fsn3.1232

**Published:** 2019-10-16

**Authors:** Qi Zhou, Xiao Jia, Qianchun Deng, Hong Chen, Hu Tang, Fenghong Huang

**Affiliations:** ^1^ Oil Crops Research Institute of the Chinese Academy of Agricultural Sciences Oil Crops and Lipids Process Technology National & Local Joint Engineering Laboratory Key Laboratory of Oilseed Processing of Ministry of Agriculture Hubei Key Laboratory of Lipid Chemistry and Nutrition Wuhan China

**Keywords:** bioactive compound degradation, canolol, oxidative stability, rapeseed oil

## Abstract

Canolol is a potential antioxidation ingredient in rapeseed oil. Rapeseed oil with two levels of canolol (528.9 vs. 250.5 mg/kg) was used for stir‐frying different foods (potatoes, tofu, and vegetables). Comprehensive evaluations indicated that the canolol content in high canolol rapeseed oil (HCR) and low canolol rapeseed oil (LCR) after stir‐frying were in the range of 187.8–237.7 and 45.6–96.4 mg/kg, respectively. The degradation rate of total phenol was 58.4% and 80.3% in HCR and LCR, respectively. The loss rates of α‐ and γ‐tocopherol were 24.5% and 47.6%, respectively. Phytosterol concentration decreased by 20% and trans‐fatty acid was not detected in either rapeseed oil. In addition, the peroxide value, anisidine value, and malondialdehyde content in HCR were lower than those in LCR. The oxidative stability index in HCR was longer, showing lower extent of deterioration. Rapeseed oil with high canolol content displayed good oxidation resistance due to significant positive correlation with oxidation induction time (*p* < .01).

## 1 INTRODUCTION

After palm oil and soy oil, rapeseed oil is the third most popular edible oil worldwide. The consumption of rapeseed oil in daily cuisine has gradually increased over the past years. Rapeseed oil is rich in plant polyphenols, phytosterols, tocopherols, and other lipid bioactive compounds. It has been implicated as a positive mediator against cardiovascular diseases and insulin resistance, making it an ideal source of vegetable oil (Wijesundera, Ceccato, Fagan, & Shen, 2010; Gawrysiak‐Witulska, Rudzińska, Siger, & Bartkowiak‐Broda, 2015; Capel et al. 2018). The main functions of edible oil in cooking are color enhancement, color protection, flavoring, and emulsification. In addition, edible oils can be considered heat energy transfer media.

Among the important components of rapeseed oil, 2,6‐dimethoxy‐4‐vinylphenol (canolol), which is a free polyphenol and a potent radical scavenger, has received considerable attention in the last few years (Rekas, Scibisz, Siger, & Wroniak, 2016; Shrestha, Stevens, & Meulenaer, 2013). Several research teams have studied the extraction, formation mechanism, antioxidant activity, and enrichment products of canolol and other free phenolic acids during rapeseed oil processing (Rekas, Wroniak, & Krygier, 2015; Siger, Czubinski, Dwiecki, Kachlicki, & Nogala‐Kalucka, 2013; Siger & Józefiak, 2016; Siger, Józefiak, & Górnaś, 2017). Canolol content in rapeseed oil can be enhanced via microwaving or roasting (Siger, Gawrysiak‐Witulska, & Bartkowiak‐Broda, 2017; Wijesundera et al., 2010). Results of in vitro antioxidative assay and deep‐frying test showed that rapeseed oil with high canolol content have better oxidative stability than that with low canolol content (Matthäus, Pudel, Chen, Achary, & Thiyam‐Holländer, 2014; Shrestha, Gemechu, & Meulenaer, 2013; Yang et al., 2014 **)**. However, researches of evaluating the quality changes in canolol‐rich rapeseed oil are lacking.

Traditional Chinese food has a long history and attractive culture. The cooking methods in China are diverse, with abundant use of high temperature processing and multi‐oil recipes. Among them, stir‐frying is the most conventional and efficient cooking method used for preparing Chinese family cuisine. For the stir‐frying process, edible oil is heated to high temperatures and mixed with other materials for a relatively short time (Cui, Hao, Liu, & Meng, 2017). Heating temperature and time differently affect the chemical composition and oxidation products of oils (Hosseini, Ghorbani, Meshginfar, & Mahoonak, 2016; Zribi et al., 2014). Vegetable oils are used for deep‐frying for preparing Western country cuisine, which mainly includes potato and chicken (Abiona, Awojide, Anifowoshe, & Babalola, 2011; Shrestha, Stevens, et al., 2013; Wakako, Akiko, & Kaori, 2010). The effects of different cooking methods on the formation of oxidative products and trans‐fatty acids profiles in various types of fish and chicken have been extensively investigated (Chen et al., 2014; Neff, Bhavsar, Braekevelt, & Arts, 2014; Turkkan, Cakli, & Kilinc, 2008). However, unsaturated fatty acids (UFAs) can be converted into trans‐fatty acids (TFAs) via thermal oxidative deterioration. Hence, cis and trans‐fatty acid composition is an important index for evaluating edible oil. Studies have mainly focused on total polar compound content, cis and trans‐fatty acid composition, oxidation characteristics, and other risk factors of fried edible oil after pan frying and deep‐frying (Aladedunye & Przybylski, 2014; Felixa & Roman, 2009; Shinde & Gupta, 2014). The influence of four different cooking methods, namely, roasting, grilling, microwaving, and frying, on cooking loss, lipid oxidation, and volatile profile of foal meat was studied by Domínguez et al. (Domínguez, Gómez, Fonseca, & Lorenzo, 2014). Recently, TFAs content in edible vegetable oil also was evaluated based on the four types of Chinese cooking methods (vegetable salad, stir‐frying, pan‐frying, and deep‐frying) (Cui et al., 2017).

The accumulation of high levels of bioactive compounds in daily diet is an important aspect to be considered while evaluating oil quality. With the rapidly increasing choices of different edible oils in the market, coupled with insufficient information on their appropriate use, selection of the best dietary oil is becoming an increasingly challenging task. To the best of our knowledge, studies discussing the effect of stir‐frying on different processed food are limited. In this study, based on the traditional Chinese stir‐frying method, rapeseed oil with two different canolol levels were used to evaluate various parameters such as fatty acid content, bioactive compound content, presence of primary and secondary oxidation products, and oxidative stability index (OSI). Our observations will provide basic data, which will assist in selecting rapeseed oil suitable for cooking.

## 2 MATERIALS AND METHODS

### 2.1 Chemicals

Solvents used for extraction and analysis included 95% HPLC‐grade n‐hexane and methanol, which were purchased from Merck Reagents Co., Ltd.. Other common analytical grade reagents, including ethanol, diethyl ether, acetic acid, chloroform, and sodium hydroxide, were obtained from Sinopharm Chemistry Reagents Co., Ltd.. Folin's‐Ciocalteu reagent (purity, >97%), 5α‐cholesterol, tocopherols (α‐, β‐, γ‐, δ‐), and eight types of trans‐fatty acids (TFAs) standards (C_14_–C_22_) were purchased from Sigma‐Aldrich Chemical Co.. Brassicasterol, campesterol, β‐sitosterol, and Δ^5^‐avenasterolwere obtained from Blue sky Biological Engineering Co., Ltd.. A mixture of fatty acid methyl ester standards was purchased from NU‐CHEK‐PREP. Canolol (purity, >98%) was purchased from BOCSCI Inc.

### 2.2 Sample preparation

High canolol rapeseed (HCR): Two hundred kilograms of rapeseed were heated in a commercial tunnel microwave oven (2,450 Hz, 55 KW) for 7 min and the whole transfer line was 10 m under the microwave machine. The microwaved rapeseeds were pressed using twin screw oil press machine (Wuhan, China), and the obtained rapeseed oil was degummed and deacidificated using physical adsorbent in order to get qualified frying oils. Low canolol rapeseed (LCR) was purchased from the factory with the hot pressing process. High canolol rapeseed (HCR) and low canolol rapeseed (LCR) were refined using physical methods.

### 2.3 Stir‐frying and oil extraction

The stir wok (diameter, 32 cm, Supor) was washed and wiped before the frying experiments. All ingredients and oils were matched according to the prescribed ratio. To minimize human error, the same operator performed the entire process. Before stir‐frying, the oils were heated within 40 s.

#### 2.3.1 Fried shredded potato

Potato (700 g) was peeled, cut in pieces, and soaked in water for 3 min. Thirty milliliters oil sample (HCR and LCR, respectively for six parallels) were put into a stir wok. The shredded potato was put into stir wok when the temperature of oil was near its smoke point. The potato in the wok was stirred for 3 min. After cooking, the shredded potato were collected and stored at −20°C until further analysis.

#### 2.3.2 Fried tofu

Tofu (600 g) was put into a stir wok with 30 ml oil sample (HCR and LCR with six parallel operations). During the process, 10 ml water was added gradually to prevent the tofu from burning. The content in the wok was stirred for 5 min. After cooking, the tofu were collected and stored at −20°C until further analysis.

#### 2.3.3 Fried vegetables

Chinese cabbage (600 g) was selected and cleaned. Chinese cabbage was put into a stir wok with 30 ml oil sample (HCR and LCR, each with six parallel operations). The content in the stir wok was stirred for 1 min. After cooking, the Chinese cabbage was collected and stored at −20°C until further analysis.

Parallel operations were conducted for six cycles, and three replicates were used for each material. At the same time, uncooked materials (potato, tofu, and Chinese cabbage) were collected and oil was extracted from them to eliminate the influence of bioactive ingredients in material oil. Oil was extracted from food and cooked oil according to Cui et al. (2017). Starting from oil heating, the temperature of the pan was monitored every 20 s using infrared detector.

### 2.4 Determination of 2,6‐dimethoxy‐4‐vinylphenol (canolol) content

Rapeseed oil sample (0.5 g) was added into a 10 ml colorimetric tube, followed by the addition of 1.5 ml hexane and 1.5 ml methanol: water (ratio, 4:1). The mixture was vortexed for 5 min at 706 *g* and centrifuged for 10 min at  2,823 *g*. The supernatant was repeat extracted twice as described above. The mixed extracts were passed through a 0.45‐µm filter. Three replicates were used per experiment. Canolol was quantified using UPLC analysis according to Yang et al. (2014).

### 2.5 Determination of oxidative index

The peroxide value, p‐anisidine value, and malonaldehyde content were measured according to the conventional method ISO 3960, ISO 6885–2006, and GB 5009.181–2009 standards, respectively.

### 2.6 Determination of cis and trans‐fatty acid composition

Methyl esterification of the cis‐ and trans‐fatty acids was performed according to Zribi et al. (2014). Cis‐ and trans‐fatty acid compositions were analyzed using the Agilent 7890N GC instrument coupled with a flame ionization detector (Agilent Technologies Inc.). A nonpolar fused‐silica capillary column (HP‐88, 100 m × 0.25 mm × 0.20 μm) was used for gas chromatography (GC) analysis. Helium was used as a carrier gas at a flow rate of 1.5 ml/min. A split ratio was set as 1:30. The temperature program was as follows: Initial temperature set at 120°C was on hold for 1 min, after which it was increased to 180°C at 20°C/min; next, the speed was set as 5°C/min, and finally, the column temperature was increased to 220°C and held for 5 min. Both the detector and injector port temperatures were maintained at 260°C. The fatty acids were identified from retention time comparison versus mixed standards run under the same conditions. They were quantified according to percentage peak area ratio. The final results were expressed as the percentages of individual fatty acids.

### 2.7 Determination of total phenol content

Rapeseed oil (0.5 g) was added into a 10 ml colorimetric tube, followed by the addition of 5 ml 95% methanol and 0.5 ml Folin's phenol reagent, and mixed for 3 min. One milliliter saturated Na_2_CO_3_ solution was added, and the mixture was diluted to a constant volume of 10 ml using distilled water. The solution was stewed and allowed to react at room temperature for 60 min. The absorbance of the sample was measured at 765 nm. The result was expressed in sinapic acid equivalent (mg/100 g oil).

### 2.8 Determination of tocopherols

Tocopherol content in rapeseed oils was determined according to Zhang et al. (Zhang et al., 2017 **)**. Different tocopherols were identified by comparing their retention times to those of α‐, β‐, γ‐, and δ‐tocopherol standards. The final result was expressed as mg/100 g.

### 2.9 Determination of phytosterols

Oil samples were saponified according to the method described by Azadmard‐Damirchi, Nemati, Hesari, Ansarin, and Fathi‐Achachlouei (2010). The 0.03 g oil sample (exact to 0.0001 g) was mixed thoroughly with 3 ml of 2 mol/L KOH in 95% ethanol in a glass tube and shaken in a water bath at 90°C for 15 min. After cooling the tubes, 2 ml of water and 1.5 ml of hexane were added and mixed vigorously. The mixture was then centrifuged at 5,000 rpm for 5 min, and the hexane layer containing unsaponifiables was separated for further analysis. The phytosterols were quantified using GC analysis according to Yang et al. (2013). The GC conditions for phytosterol identification are as follows: DB‐5 capillary column (30.0 m × 320 μm × 0.10 μm) was used for analysis; the injector temperature was 260°C; the temperature program was as follows: hold at 60°C for 1 min, increased at 40°C/min to 310°C, and maintained for 6 min; the carrier gas was helium with flow rate of 2 ml/min; and analysis was performed in a split ratio of 10:1.

### 2.10 Oxidation induction time

The OSI of oil samples was evaluated using a Metrohm 743 Racimat apparatus (Metrohm Herisan, Switzerland). The oil samples (3.0 g) were heated at 110°C. This method is based on the hypothesis that continuous bubbling of air through the oil‐containing sample at constant speed of 20 L/h can be used to measure the oxidation induction time of the oil. Results were expressed as hours.

### 2.11 Statistical analysis

Analysis of variance (ANOVA) and Duncan's multiple range tests were performed using the SPSS (Statistical Product and Service Solutions) 19.0 software version. The ANOVA test was performed for all experimental runs to determine the significance at 95% confidence interval. All experiments were performed in quintuplicate. The means and standard deviations for all values were computed in Origin 8.0.

## 3 RESULTS AND DISCUSSION

### 3.1 Characterization of primary and secondary oxidation products after stir‐frying

**Figure 1:**
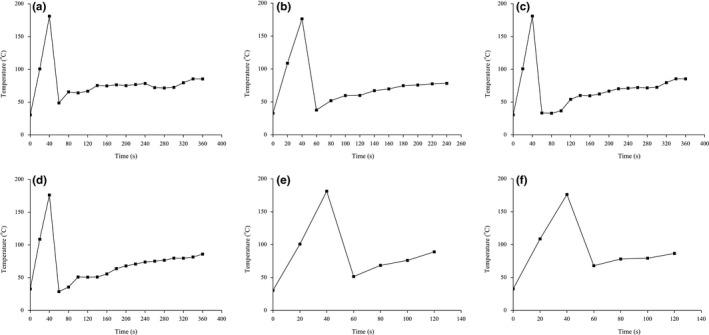
The temperature changes during stir‐frying. HCR‐P (a), LCR‐P (b), HCR‐T (c), LCR‐T (d), HCR‐V (e), LCR‐V(f). HCR‐P: Extracted oil from potato with HCR; HCR‐T: Extracted oil from tofu with HCR; HCR‐V: Extracted oil from vegetable with HCR; LCR‐P: Extracted oil from potato with LCR; LCR‐T: Extracted oil from tofu with LCR; LCR‐V: Extracted oil from vegetable with LCR; Each value represents the mean of three determinations (*n* = 3) (standard deviation)

The temperature was monitored and shown in Figure 1. The oils were heated from 32 ℃ to about 190°C in 40 s. The temperature reduced rapidly when the food items were put into stir wok and then increased in the whole stir‐frying process. Peroxide value is an important indicator reflecting the primary oxidation products of oil. Table 1 shows that peroxide values ranged from 2.1 mmol/kg to 2.7 mmol/kg, 2.3 mmol/kg, and 2.3 mmol/kg in HCR from shredded potato, tofu, and vegetable, respectively, indicating a slightly increasing trend. Peroxide values ranged from 1.6 mmol/kg to 2.7 mmol/kg, 2.5 mmol/kg, and 1.9 mmol/kg in the LCR of the above cooked foods, respectively. The oxidative rancidity of oils and the deterioration of the products are affected not only by the saturation degree of the oil, but also by other factors, such as temperature, light, oxygen, metal ions, water activity, and antioxidants. The high temperature of deep‐frying is sufficient to disrupt the C‐C and C‐H covalent bonds of the acyl skeleton, forming various lipid hydrocarbyl radicals, which initiates the chain reaction of free radical oxidation (Goyary, Kumar, & Nayak, 2015). However, after a short stir‐frying, the peroxide value was less than 6 mmol/kg, which was within the range of the recommended standard for oil. The effect of quick stir‐frying on peroxide value is limited.

**Table 1:** Oxidation extent of rapeseed oils before and after stir‐frying

Oxidation index	HCR	HCR‐P	HCR‐T	HCR‐V	LCR	LCR‐P	LCR‐T	LCR‐V
Peroxidevalue (mmol/kg)	2.1 ± 0.3^a^	2.7 ± 0.1^b^	2.3 ± 0.1^a^	2.3 ± 0.2^a^	1.6 ± 0.1^a^	2.7 ± 0.3^b^	2.5 ± 0.1^b^	1.9 ± 0.1^a^
Anisidine value	3.2 ± 0.1^a^	5.6 ± 0.3^b^	6.5 ± 0.4^b^	6.3 ± 0.3^b^	5.5 ± 0.3^a^	11.1 ± 0.5^b^	16.2 ± 0.4^c^	8.4 ± 0.9^d^
Malonaldehyde (μg/kg)	3.4 ± 0.2^a^	3.6 ± 0.2^a^	3.9 ± 0.2^a^	6.3 ± 0.3^b^	3.4 ± 0.2^a^	4.4 ± 0.3^b^	6.7 ± 0.5^c^	8.2 ± 0.3^d^
OSI(h)	11.8 ± 0.6^a^	9.2 ± 0.4^b^	9.4 ± 0.3^b^	7.3 ± 0.6^c^	9.5 ± 0.2^a^	4.5 ± 0.2^b^	7.6 ± 0.3^c^	3.2 ± 0.2^d^

Note

Different lower case letters (a, b, c, and d) within the same row was the Duncan's multiple range tests carried out by using SPSS.

Abbreviations: HCR, uncooked rapeseed oil with high canolol content (528.9 mg/kg); HCR‐P, Extracted oil from potato with HCR; HCR‐T, Extracted oil from tofu with HCR; HCR‐V, Extracted oil from vegetable with HCR; LCR, uncooked rapeseed oil with low canolol content (250.5mg/kg); LCR‐P, Extracted oil from potato with LCR; LCR‐T, Extracted oil from tofu with LCR; LCR‐V, Extracted oil from vegetable with LCR; OSI, oxidative stability index.

Oil is further decomposed to produce a series of aldehydes, ketones, and acids (O’Keefe and Pike 2010). The p‐anisidine value is an index of the nonvolatile aldehyde content in oil, formed from fatty acid hydroperoxides, especially 2‐alkenals and 2, 4‐dienals. p‐anisidine value and malondialdehyde (MDA) content is measures of the oxidative state of edible oil. P‐anisidine value and MDA content was lower in HCR than in LCR. Table 1 shows that p‐anisidine value increased highly and significantly (*p* < .05) from 3.2 to 6.5 and from 5.5 to 16.2 for HCR and LCR, respectively. The extent of increase was different for HCR and LCR, which may be because of their initial levels in uncooked oils. P‐Anisidine value of more than 10 indicates severe deterioration (Neff et al., 2014 **)**. The canolol, total phenol, and total phytosterol contents showed significant correlation with the p‐anisidine value of HCR samples (*p* < .05, *r* > .9). Similarly, Matthaus et al. (Matthäus et al., 2014) observed p‐anisidine values of oil with different concentrations canolol were monitored in the range from 2 to 40 after frying 6 hr, with highly negative linear correlation between the amount of added antioxidant and the p‐anisidine value. However, no significant correlation between the bioactive compound level and p‐anisidine value was observed in LCR.

MDA causes skin cancer in rats as it cross‐links with amino groups of DNA. MDA can damage proteins or phospholipids via covalent bonding and cross‐linking. Frying in oil that contains mutagens can lead to production of hazardous compounds. The MDA content is the measure of the degree of peroxidation of the plant cytoplasmic membrane. High MDA content is indicative of the high degree of peroxidation and serious damage to the plant cell membrane. MDA content of HCR and LCR increased from 3.4 μg/kg to maximum 6.3 μg/kg and 8.2 μg/kg (*p* < .05), respectively, after stir‐frying (Table1). The increase for the LCR samples was higher than that for the HCR samples, which indicated that HCR suffered less oxidative deterioration after stir‐frying. Lower p‐anisidine value and MDA content in oil indicated lower extent of oxidation.

### 3.2 Changes in fatty acid composition after stir‐frying

Heating can significantly affect the chemical composition of oils. The presence of unsaturated fatty acids (UFAs) in vegetable oils, including monounsaturated fatty acids (MUFAs) and polyunsaturated fatty acids (PUFAs), is associated with decreased risk of coronary heart disease (Bendsen, Christensen, Bartels, & Astrup, 2011).

The most predominant fatty acids in uncooked rapeseed oil are palmitic acid (4.20% and 5.31%), oleic acid (58.97% and 58.22%), linoleic acid (17.35% and 20.79%), linolenic acid (8.18% and 7.65%), arachidonic acid (3.89% and 2.12%), and erucic acid (5.18% and 3.55%) (Table 2). The results of this study are in agreement with those reported previously, which have shown that the major fatty acids in rapeseed oil included 5.9% of C16:0, 2.5% of C18:0, 48.4% of C18:1, 29.4% of C18:2, and 8.8% of C18:3 (Marinova et al., 2001). Significant differences (*p* < .05) were identified between the oils using multiple comparison analysis. TFAs were not detected after stir‐frying, and simultaneously, no significant differences were observed in the level of fatty acids among the rapeseed oils. Linolenic acid content decreased after cooking from 8.18% to 7.97% in HCR and from 7.65% to 7.47% in LCR, respectively. Results of other studies showed that the linoleic acid content decreased progressively by 8.5% and 13.3% during frying at 185°C and 215°C, respectively (Felixa & Roman, 2009 **)**. The deterioration of linolenic acid content was more pronounced and was reduced by 24.0% and 47.1% during frying at 185°C and 215°C, respectively (Felixa & Roman, 2009 **)**. When the relative percentages of fatty acids were determined after stir‐frying, a slight decrease (not significant, *p* > .05) of UFA content was observed (Table 2) due to low temperature. A considerable decrease was observed in the PUFA content, including those of linoleic and linolenic acids. Indeed, the amount of C18:1 slightly but significantly increased (*p* < .05) for all samples. According to Enríquez‐Fernández et al. (2011) and Romero, Cueta, and Sánchez‐Muniz (2000), the slight increase in oleic acid content might be because of linoleic and linolenic acid degradation. The C18:2/C16:0 ratio decreased for both HCR and LCR samples (Table 2) from 4.13% to 4.03% and from 3.94% to 3.87%, respectively. In fact, linoleic acids are usually used as indicators of the extent of fat deterioration because it is more susceptible to oxidation than saturated fatty acids. Therefore, the C18:2/C16:0 ratio is also used to indicate the degree of oxidative deterioration in frying oil (Flakelar et al. 2017 **)**. Overall, our results indicate that the oxidation resistance was higher in HCR than in LCR.

**Table 2:** Fatty acid compositions of rapeseed oils before and after stir‐frying

FA content (% total FA)	HCR	HCR‐P	HCR‐T	HCR‐V	LCR	LCR‐P	LCR‐T	LCR‐V
C16:0	4.20 ± 0.02^a^	4.23 ± 0.02^a^	4.22 ± 0.02^a^	4.25 ± 0.00^b^	5.31 ± 0.05^a^	5.37 ± 0.01^a^	5.38 ± 0.05^a^	5.37 ± 0.05^a^
C16:1	0.19 ± 0.00^a^	0.20 ± 0.00^a^	0.20 ± 0.00^a^	0.20 ± 0.00^a^	0.20 ± 0.00^a^	0.20 ± 0.00^a^	0.20 ± 0.00^a^	0.20 ± 0.00^a^
C18:0	2.03 ± 0.01^a^	2.06 ± 0.02^a^	2.04 ± 0.02^a^	2.07 ± 0.01^a^	1.98 ± 0.02^a^	1.99 ± 0.02^a^	1.98 ± 0.01^a^	1.98 ± 0.02^a^
C18:1	58.97 ± 0.05^a^	59.21 ± 0.11^b^	59.14 ± 0.10^b^	59.15 ± 0.15^b^	58.22 ± 0.07^a^	58.55 ± 0.20^c^	58.35 ± 0.16^b^	58.35 ± 0.20^b^
C18:2	17.35 ± 0.03^a^	17.21 ± 0.03^b^	17.31 ± 0.04^a^	17.15 ± 0.06^b^	20.97 ± 0.07^a^	20.79 ± 0.04^b^	20.86 ± 0.13^b^	20.83 ± 0.02^b^
C18:3	8.18 ± 0.04^a^	7.97 ± 0.02^b^	8.03 ± 0.02^c^	8.01 ± 0.01^c^	7.65 ± 0.04^a^	7.47 ± 0.01^b^	7.58 ± 0.03^c^	7.56 ± 0.02^c^
C20:1	3.89 ± 0.06^a^	3.91 ± 0.08^a^	3.92 ± 0.03^a^	3.96 ± 0.03^a^	2.12 ± 0.03^a^	2.16 ± 0.07^a^	2.14 ± 0.03^a^	2.17 ± 0.03^a^
C22:0	5.18 ± 0.08^a^	5.20 ± 0.02^a^	5.13 ± 0.12^a^	5.22 ± 0.10^a^	3.55 ± 0.11^a^	3.46 ± 0.13^a^	3.52 ± 0.11^a^	3.54 ± 0.22^a^
∑SFA	11.41 ± 0.11^a^	11.49 ± 0.04^a^	11.39 ± 0.16^a^	11.54 ± 0.11^a^	10.84 ± 0.18^a^	10.82 ± 0.16^a^	10.88 ± 0.17^a^	10.89 ± 0.32^a^
∑PUFA	25.53 ± 0.07^a^	25.18 ± 0.19^b^	25.34 ± 0.06 ^b^	25.16 ± 0.07^b^	28.62 ± 0.11^a^	28.26 ± 0.05^b^	28.44 ± 0.16^b^	28.39 ± 0.04^b^
∑MUFA	63.05 ± 0.11^a^	63.33 ± 0.19^b^	63.27 ± 0.13^a^	63.30 ± 0.18^a^	60.54 ± 0.10^a^	60.92 ± 0.27^c^	60.68 ± 0.19^b^	60.72 ± 0.23^b^
∑UFA	88.58 ± 0.18^a^	88.51 ± 0.38^a^	88.61 ± 0.19^a^	88.46 ± 0.25^a^	89.16 ± 0.21^a^	89.18 ± 0.32^a^	89.12 ± 0.35^a^	89.11 ± 0.27^a^
C18:2/C16:0	4.13	4.07	4.10	4.03	3.94	3.87	3.87	3.87

Abbreviations: MUFA, monounsaturated fatty acids; PUFA, polyunsaturatedatty acids; SFA, saturated fatty acids; UFA, unsaturated fatty acids.

Different lower case letters (a, b, and c) within the same row was the Duncan's multiple range testscarried out by using SPSS

TFAs are always used for evaluating the quality of frying oil. With the exception of cis fatty acid, TFAs were not detected after comparison with standards poststir‐frying. TFAs were not detected in any sample (Table 2). The TFAs concentration in frying oils can be determined from the extent of partial oil hydrogenation. The amounts of trans C18:1 in the fresh canola oils were less than the quantitative limit (QL). They were lesser than QL even after the tenth frying operation at 160, 180, and 200°C (Wakako et al., 2010). Trans C18:2 fatty acid accumulation in frying oil was statistically different from that in heated oil. High temperature (190°C) can increase the amount of trans‐isomers during deep‐frying in edible oils (Zribi et al., 2014 **)**. Cui et al. showed that the TFAs content in rapeseed oil with control sample (8.26) was slightly lesser than that of vegetable salad oil (8.33), stir‐frying oil (10.76), and pan‐frying oil (9.28) (Cui et al., 2017 **)**. TFAs were not formed in any of the samples in our study, possibly because the temperature of stir‐frying did not reach the condition at which TFAs are produced, or the time of frying was not sufficient.

### 3.3 Degradation of bioactive components in the rapeseed oils after stir‐frying

**Figure 2:**
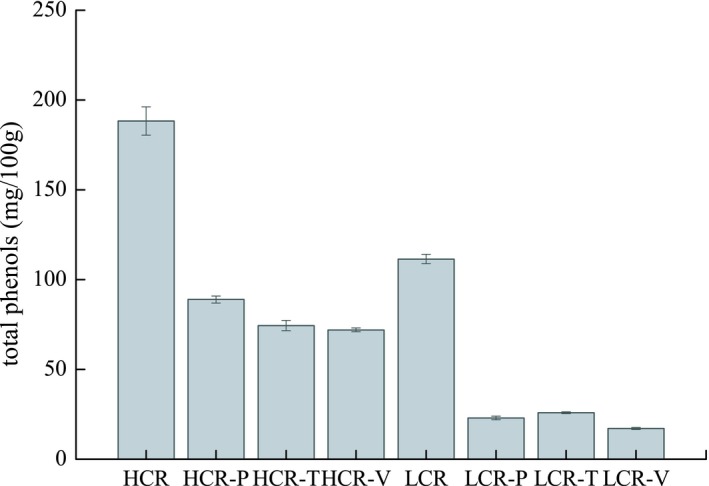
Total phenols level of rapeseed oils before and after stir‐frying. HCR: uncooked rapeseed oil with high canolol content (528.9 mg/kg); LCR: uncooked rapeseed oil with low canolol content (250.5mg/kg)

Internal bioactive compounds or exogenous antioxidants are used to improve the thermal stability of oil during processing as they can inactivate pro‐oxidant metals, scavenge free radicals, or quench singlet oxygen depending on their functional group. The total phenol and canolol level of rapeseed oil was increased by microwaving or roasting, as detected in the sample preparation obtained from an oil factory. Oil stability during food processing can be easily determined from the performance of a frying experiment and analysis of relevant parameters associated with the quality of the used frying oil. The initial content of total phenols in HCR and LCR oils was 188.3 mg/100 g and 111.4 mg/100 g, respectively (Figure 2). After frying potatoes, the content of total phenols decreased to 88.9 mg/100 g and 23.0 mg/100 g, respectively, with a percentage of weight loss of 53.2 and 79.3%, respectively. The total polyphenols of HCR and LCR were reduced to 72.0 and 17.1 mg/100 g, respectively, during the frying of vegetables (Figure 2) and the percentage weight loss were 61.7% and 84.6%, respectively. While frying tofu, the content of total phenols decreased to 74.4 and 25.9 mg/100 g, respectively, with a percentage weight loss of 60.4% and 76.7%. Therefore, after frying three raw materials, the total phenol content of HCR was 70.5–88.9 mg/100 g, whereas for LCR it was of 17.1–25.9 mg/100 g. It is obvious that the total phenol concentration of HCR was significantly higher than that of LCR.

**Figure 3:**
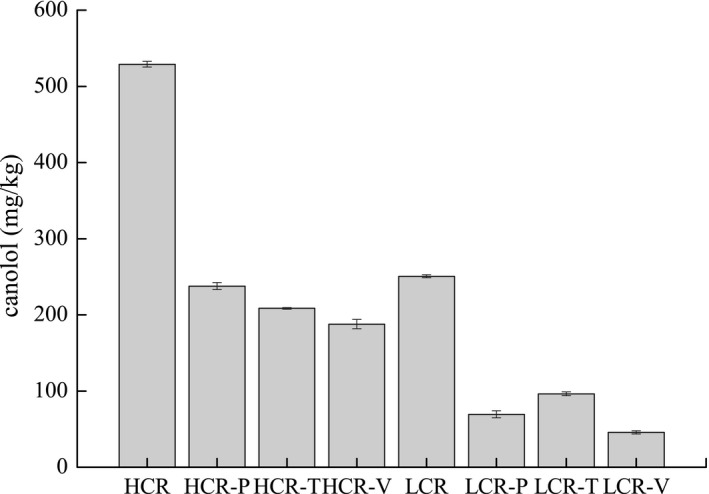
Canolol level of rapeseed oils before and after stir‐frying

2,6‐Dimethoxy*‐*4‐vinylsyringol is a newly identified phenolic compound in rapeseed oil that is formed by thermal decarboxylation of the sinapic acid naturally occurring in rapeseed seeds after roasting or microwaving (Yang et al., 2014 **)**. Thermal treatment of rapeseed increased the canolol content, and temperature considerably influenced the amount of canolol generated in rapeseed. However, it may be converted into polymeric canolol or be degraded during cooking. During potato frying, the level of canolol in HCR and LCR decreased from 528.9 to 237.7 mg/kg and from 250.5 to 69.4 mg/kg, respectively, with a loss rate of 55.0% and 72.3%, respectively (Figure 3). The degradation rate of canolol was 64.5% for HCR and 81.8% for LCR in tofu frying and 60.5% for HCR and 61.5% for LCR in vegetable frying, respectively. Therefore, the level of canolol in HCR after frying different raw materials was in the range of 187.8–237.7 mg/kg, with a loss of 55–64.5%. The percentage of weight loss of canolol in LCR was in the range of 61.5–81.8%, while the concentration after cooking was in the range of 45.6–96.4 mg/kg. Canolol content correlated significantly with induction time for both HCR and LCR. It correlated significantly with p‐anisidine value in HCR and with MDA in LCR. Interestingly, Matthäus et al. (2014) obtained a similar result, showing that the degradation rate of canolol decreased with increase in the initial concentration of canolol‐enriched extracts Hosseini et al. (2016) found lower content in the oil at higher frying temperature.

There are four forms of tocopherols in rapeseed oil, including α‐, β‐, γ‐, and δ‐tocopherols. Tocopherols play an important role as antioxidants, as they prevent oxidation reactions by donating hydrogen atoms to peroxyl radicals (Normand, Eskin, & Przybylski, 2001; Saguy & Dana, 2003 **)**. Two types of tocopherols, α‐tocopherol and γ‐tocopherol, were detected in the two rapeseed oils. The main isomer of tocopherol in rapeseed oil is γ‐tocopherol, which accounts for almost two‐third of the total amount of tocopherol. These preliminary observations are consistent with the results of previous studies showing that α‐tocopherol (130 mg/kg) and γ‐tocopherol (320 mg/kg) were detected in raw rapeseed oil (Barrera‐Arellano, Ruiz‐Mendez, Velasco, Marquez‐Ruiz, & Dobarganes, 2002).

**Figure 4:**
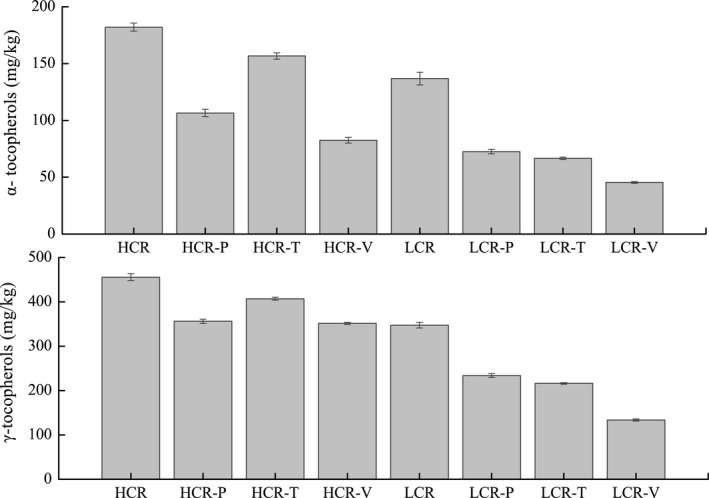
α‐tocopherols and γ‐tocopherols level of rapeseed oils before and after stir‐frying

Figure 4 shows that the contents of α‐ and γ‐tocopherols were 182.1 and 455.5 mg/kg in HCR and 136.9 and 347.5 mg/kg in LCR. After vegetable frying, α‐, and γ‐, tocopherol content decreased to 82.5 and 351.3 mg/kg in HCR and to 45.4 and 133.7 mg/kg in LCR, respectively. Results suggested the content of both α‐ and γ‐tocopherols decreased significantly after frying different raw materials, although the content of α‐ and γ‐tocopherols in HCR was significantly higher than those in LCR. The γ‐tocopherol content of fresh canola oil used was 214.0 mg/kg, which was similar to the 347.0 mg/kg of tocopherol reported by Felixa and Roman (2009). We also observed that approximately 31% of the total tocopherols remained after frying at 185°C. Loss in natural tocopherol content in oils with different degrees of unsaturation, heated via the two heating periods to 180°C, ranged from 450 mg/kg to 359 mg/kg and to 145 mg/kg after 10 cycles of heating (Barrera‐Arellano et al., 2002). Compared to that after baking or boiling, the instability of tocopherols after stir‐frying is possibly because of the heat‐sensitive nature of the tocopherols (Saguy & Dana, 2003 **)**. The rates of degradation of α‐tocopherols and γ‐tocopherols were more than 40% during stir‐frying of three types of dishes. The decrease in tocopherol content was accompanied by increase in MDA in LCR (*p* < .001); however, MDA levels did not correlate with the change in tocopherols in HCR.

Phytosterols are important bioactive compounds of vegetable oils after tocopherols and phenol. Extensive studies have shown that phytosterols are involved in preventing atherosclerosis. Increasing phytosterol consumption is an effective way of containing hypercholesterolemia as it decreases cholesterol concentration in the blood.

Table 3 shows a progressive decrease in the content of phytosterols in HCR and LCR after frying of three different raw materials. The total amount of phytosterols in those two raw oils was 597.24 and 427.44 mg/100 g, respectively. The total values were 480.57 ~ 496.05 mg/100 g and 350.1 ~ 361.51 mg/100 g after stir‐frying in HCR and LCR, respectively, suggesting that the extent of phytosterol loss was lower than those of total phenols, canolol, and vitamin, with about 80% retention rate. Loss of phytosterols during stir‐frying is less, which may be due to its excellent stability at high temperature compared to that of canolol, phenols, and tocopherols. In addition, the phytosterols in rapeseed oil mainly include brassicasterol, campesterol, β‐sitosterol, and Δ5‐avenastrerol. Among these, β‐sitosterol is the most abundant, followed by campesterol, and Δ5‐avenasterol is the least abundant in HCR and LCR. β‐Sitosterol loss was about 16% after stir‐frying in both HCR and LCR. A similar degradation rate was observed in these two rapeseed oils. This may be attributed to the fact that many important components exhibit antioxidant activity during high temperature cooking. According to Rudzińska, Korczak, and Wąsowicz (2005), the oxidation products of campesterol, stigmasterol, and β‐sitosterol include epimers of 7‐hydroxy, 5,6‐epoxy, and 7‐ketotriols. Previous studies have shown that the content of phytosterols decreased significantly during deep‐frying of French fries in rapeseed oil (ca. 60%) (Marinova et al., 2012).

**Table 3:** Phytosterols in rapeseed oils before and after stir‐frying

Phytosterols (mg/100g)	HCR	HCR‐P	HCR‐T	HCR‐V	LCR	LCR‐P	LCR‐T	LCR‐V
Brassicasterol	66.87 ± 0.90^a^	53.18 ± 0.34^b^	53.77 ± 0.30^b^	54.46 ± 0.65^c^	39.38 ± 0.43^a^	33.48 ± 0.76^b^	33.09 ± 0.79^b^	32.81 ± 0.54^b^
Campesterol	193.08 ± 3.50^a^	160.57 ± 5.43^b^	155.32 ± 3.55^b^	156.70 ± 3.65^b^	137.55 ± 4.65^a^	116.91 ± 3.89^b^	115.45 ± 4.01^b^	112.72 ± 5.77^b^
β‐sitosterol	322.93 ± 3.76^a^	273.70 ± 4.55^b^	261.92 ± 2.70^c^	267.20 ± 6.33^b^	239.76 ± 3.54^a^	201.37 ± 3.44^b^	204.48 ± 0.65^b^	195.84 ± 4.32^c^
Δ5‐avenasterol	14.36 ± 0.45^a^	8.60 ± 0.77^b^	9.56 ± 0.34^c^	5.36 ± 0.59^d^	10.75 ± 0.16^a^	8.79 ± 0.65^b^	8.49 ± 0.34^b^	8.73 ± 0.23^b^
Total	597.24 ± 8.61^a^	496.05 ± 11.09^b^	480.57 ± 6.89^b^	483.71 ± 11.22^b^	427.44 ± 8.78^a^	360.55 ± 8.74^b^	361.51 ± 8.04^b^	350.10 ± 10.86^b^

Different lower case letters (a, b, c, and d) within the same row was the Duncan's multiple range testscarried out by using SPSS

### 3.4 Relationship between OSI and bioactive compounds

Comparison of micronutrients showed that the canolol, total phenolic, and phytosterol contents in HCR were significantly higher than those in LCR before and after cooking different raw materials. The degradation rate indicated slight differences in oxidation stability among the three raw materials. Among bioactive compounds with antioxidant properties, the total phenolic content showed significant (*p* < .05) differences, with initial concentrations of 188.3 mg/100 g for HCR and 111.4 mg/100 g for LCR. Consequently, the oxidative stability, as determined using the Rancimat method, also differed significantly (*p* < .05), with 11.8 hr for HCR and 9.5 hr for LCR (Table 1). Oxidative induction time decreased sharply after stir‐frying vegetables, being 7.3 hr and 3.2 hr for HCR and LCR, respectively. Analysis of the correlation between different active components of rapeseed oil, p‐anisidine value, MDA content, and oxidation induction time showed that 2,6‐dimethoxy‐4‐vinylphenol correlated significantly with oxidation induction time. The oxidative stability of oils and fats depends not only on their fatty acid composition, but also on the presence of fatty bioactive constituents, such as phytosterols and phenols (Rudzińska et al., 2005). Generally, HCR showed better cooking oxidation stability than LCR. This is similar to the results of a recent study showing that the canolol‐rich extracts were able to retard the degradation rate of α‐ and γ‐tocopherol during frying (Hosseini et al., 2016 **)**.

However, the results of correlation analysis were different in the two rapeseed oils (Table 4). In HCR samples, total phenols, canolol, total tocopherols, and phytosterols showed a significant positive relationship with OSI. Although total phenols, canolol, and phytosterols showed significant negative correlation with p‐anisidine value, they showed significant correlation with MDA content in LCR. Bioactive compounds contribute significantly to oil oxidation stability. Anisidine is formed from fatty acid hydroperoxides, especially 2‐alkenals and 2,4‐dienals. However, MDA is a degradation product of polyunsaturated fatty acid peroxide. The MDA in fried food products is mainly derived from oil oxidation. Longer duration of frying substantially increased MDA level at 170°C (Normand et al., 2001). In LCR, the total tocopherol content correlated significantly with MDA levels, but not in HCR. Canolol possess stronger antioxidant properties than tocopherols (Shrestha, Stevens, et al., 2013). In the rapeseed oils with high canolol content, canolol as a major antioxidant plays an important role in frying‐associated oxidation resistance. The good correlation with oxidative induction time illustrates its antioxidative character.

**Table 4:** Relationship between oxidative products, oxidation induction time and bioactive compounds

	Anisidine value	Malonaldehyde	OSI
HCR samples			
canolol	*r* = −.959**	*p* > .05	*r* = .874**
total phenols	*r* = −.962**	*p* > .05	*r* = .871**
total tocopherols	*p* > .05	*r* = −.728**	*r* = .873**
total phytosterols	*r* = −.949**	*p* > .05	*r* = .840**
LCR samples			
canolol	*p* > .05	*r* = −.728**	*p* > .05
total phenols	*p* > .05	*r* = −.720**	*p* > .05
total tocopherols	*p* > .05	*r* = −.893**	*p* > .05
total phytosterols	*p* > .05	*r* = −.730**	*p* > .05

** Correlation is significant at the .01 level.

## 4 CONCLUSION

During stir‐frying, plant oils are heated at high temperatures and mixed with food ingredients in a relatively short period of time. Hence, it was necessary to evaluate the changes in rapeseed oil quality in conventional stir‐frying style of cooking. In summary, oils with two different canolol contents, HCR and LCR, were studied with respect to oxidation stability. Results showed that oils with high micronutrient content manifested higher oxidation stability. When frying different raw materials (vegetable, tufu, potato), the contents of total phenols, phytosterols, and 2,6‐dimethoxy‐4‐vinylphenol in HCR were significantly higher than those in LCR, in addition to lower cooking degradation rates, indicating that HCR showed better antioxidant capacity during frying. Statistically significant differences (*p* < .05) between HCR and LCR were observed for bioactive compound concentrations and OSI. Therefore, high‐quality rapeseed oil is suitable for cooking different raw materials due to its good oxidation stability during cooking and high content of micronutrients in oil after stir‐frying. We concluded that HCR oils are advantageous for Chinese stir‐frying as they provide nutritional and health benefits.

## CONFLICT OF INTEREST

There is no conflict of interest in this paper.

## ETHICAL APPROVAL

All authors were actively involved in the work leading to the manuscript and will hold themselves jointly and individually responsible for its content. There is no conflict of interest in this paper. Human or animal testing is unnecessary in our study.
